# Role of mTOR Complexes in Neurogenesis

**DOI:** 10.3390/ijms19051544

**Published:** 2018-05-22

**Authors:** Francesca LiCausi, Nathaniel W. Hartman

**Affiliations:** Biology Program, School of Natural Sciences and Mathematics, Stockton University, Galloway, NJ 08205, USA; nams@stockton.edu

**Keywords:** mTOR, neural stem cells, differentiation, dendrite, synapse formation

## Abstract

Dysregulation of neural stem cells (NSCs) is associated with several neurodevelopmental disorders, including epilepsy and autism spectrum disorder. The mammalian target of rapamycin (mTOR) integrates the intracellular signals to control cell growth, nutrient metabolism, and protein translation. mTOR regulates many functions in the development of the brain, such as proliferation, differentiation, migration, and dendrite formation. In addition, mTOR is important in synaptic formation and plasticity. Abnormalities in mTOR activity is linked with severe deficits in nervous system development, including tumors, autism, and seizures. Dissecting the wide-ranging roles of mTOR activity during critical periods in development will greatly expand our understanding of neurogenesis.

## 1. Introduction

Neurogenesis from the embryonic brain throughout adulthood requires a well-regulated interaction of inductive cues and secreted signals that guide the development of neural stem cells (NSCs). For a stem cell to transition into a properly functioning neuron it must process complex external and internal signals to divide, differentiate, migrate, and integrate properly. These stages are guided by extrinsic factors that promote the transcription of genes to define the cell through maturation. Many extracellular signals converge on the mammalian target of rapamycin (mTOR) pathway, which is a chief regulator of cell growth, proliferation, and protein translation. Several studies have shown that mTOR is involved in many aspects of neurogenesis. Activation of mTOR alters NSC differentiation, neural progenitor migration, dendrite development, and neuron maturation.

Disruption in mTOR pathway activity leads to a host of neurodevelopmental disorders, including the congenital multisystem disorder tuberous sclerosis (TS) [1,2,3]. Cortical malformations form in most TS patients during fetal and early postnatal development, and they contribute to the development of seizures and cognitive deficits [4,5]. More than 50% of TS patients develop varying degrees of Autism Spectrum Disorder (ASD) [6]. ASD patients exhibit impaired social interactions, repetitive behaviors, and diminished interests. Hyperconnectivity of neuronal circuits by the disruption in protein synthesis is thought to drive these altered behaviors [7]. As ASD is comorbid with TS, hyperactivity of the mTOR pathway is strongly implicated in driving both pathologies. Studies in mouse models of autism that alter the upstream elements of the mTOR pathway recapitulate cortical malformations [8,9,10,11]. In addition, many cortical neurons exhibit enlarged dendrites, altered dendritic spines, and hyperactivity, which can result in seizure episodes [12,13,14]. Additionally, increasing mTOR activity in neurons can result in ASD-like behaviors in mice [15]. Because mutations upstream of the regulators of the mTOR pathway in NSCs lead to hyperactivity in mature neurons, it is important to understand mTOR signaling during nervous system development. In this review, we describe our current understanding of the role of mTOR and related pathways at each stage of neurogenesis, focusing on embryonic cortical and postnatal development.

## 2. Overview of Cortical and Postnatal Neurogenesis

NSCs arise from the ectoderm with their fate being influenced by the timely activation of transcription factors and development mediated through the Akt/mTOR pathway. As development of the neural tube progresses, the rostral regions enlarge to form three primary vesicles, including the early prosencephalon [16]. The population of NSCs from the anterior neural tube develops into multipotent radial glia progenitors in the telencephalic vesicle. These divide to form radial glial cells (RGCs) at the most apical layer of the ventricular zone.

The majority of corticogenesis occurs in rodents from embryonic day 10.5 to embryonic day 18.5 where RGCs give rise to the majority of excitatory neurons in the cerebral cortex [16]. Primary divisions of RGCs are asymmetric and form a new RGC and intermediate progenitor. With continued development, intermediate progenitors of RGCs migrate from the apical ventricular zone and comprise the subventricular zone (SVZ), which is a second zone of proliferation [17]. Intermediate progenitors migrate along the apical processes of RGCs, delaminate, and begin to form the cortical plate where they continue to differentiate into post-mitotic glutamatergic neurons. Each cortical layer is produced sequentially in an inside-out manner where progenitors that are produced earlier populate the deepest layers of the cortex and neurons that will be in superficial layers are produced later [16]. In addition, NSCs in the ventral ganglionic eminences produce progenitors that migrate tangentially into the developing cortex where they establish the network of inhibitory interneurons [18,19]. Upon reaching their final location in the cortex, both types of neurons begin to extend dendrites, as well as a single axon in order to form synaptic connections necessary for mature neural circuits.

The postnatal SVZ is a transient state in the first week of postnatal life, during which the embryonic SVZ transitions to the adult SVZ [20,21]. RGCs begin to retract their apical processes that contact the pia, and their rates of proliferation slow [22,23]. Neurogenesis in the SVZ is a tightly regulated event where the NSCs expand their population and differentiate into neuroblasts that incorporate into developing circuits [24]. NSCs contact the lateral ventricles and blood vessels in the SVZ exposing them to important guidance molecules, such as IGF-1 and EGF, which can influence the downstream signaling cascades, like Akt and mTOR [25]. NSCs differentiate into daughter cells, called transit amplifying cells (TACs), which are highly proliferative. TACs, in turn, differentiate into neuroblasts that then migrate through the SVZ, into the rostral migratory stream (RMS) and become mature neurons in the olfactory bulb (OB). In addition, postnatal NSCs contribute to both neuron and glia populations, with the majority of newly born neurons migrating to the OB [26,27,28]. Newly born neurons mature and form connections that are stereotypic of their position in the OB. For example, newly born granule neurons extend a single apical dendrite that branch in the external plexiform layer to form dendro-dendritic connections with mitral cells. These cells form extensive dendritic arbors in the first three weeks of their development before pruning dendrites and spines as they mature [29].

## 3. The mTOR Signaling Network

**Figure 1:**
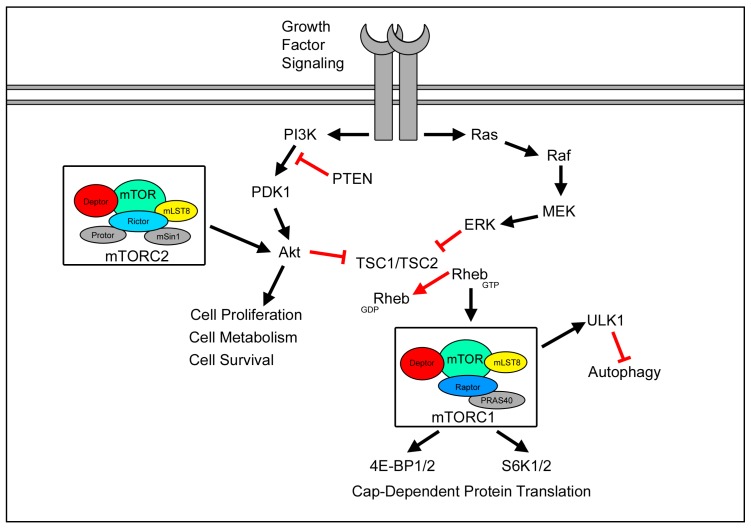
Overview of the mammalian target of rapamycin (mTOR) signaling pathway. mTOR signaling is activated in several ways, including growth factors, amino acids, and increased cellular energy. Growth factors can activate both mTOR complexes or PI3K and ERK signaling. mTORC2 phosphorylates Akt at S473 to enhance activation. In turn, Akt suppress TSC2, which suppresses Rheb. Additionally, ERK can inhibit TSC2 to promote mTORC1 activation. Phosphorylation of 4E-BPs and S6K1/2 by mTORC1 promote cap-dependent translation.

Increasing protein synthesis in responses to growth and proliferation is essential to maintain homeostasis in all cell types. mTOR is a large serine/threonine protein kinase that is a part of the phosphoinositide 3-kinase (PI3K)-related kinase family with two divergent complexes, mTOR complex 1 (mTORC1) and 2 (mTORC2). mTORC1 integrates highly intracellular ATP, glucose, and certain amino acid signaling to regulate cellular processes (Figure 1). Low ATP levels indirectly inhibit mTORC1 by promoting TSC1/2 complex formation [30]. The TSC1/TSC2 complex normally suppresses the GTPase Rheb [31]. GTP-bound Rheb then activates mTORC1, which is a complex containing mTOR, Raptor, MLST8, and PRAS40 [32,33]. In addition to PI3K/ Akt activation, Ras-MAPK signaling can activate mTORC1 through the phosphorylation of TSC2 and PRAS40 [34,35,36]. Activation of mTORC1 results in increased protein translation by phosphorylating 4E-BP (including 4E-BP1/2/3) and p70 S6 kinases [37,38]. 4E-BPs and S6K1/2 inhibit the formation of 5’ translation initiating complex by binding eIF-4E. mTORC1-mediated phosphorylation of 4E-BPs and S6K1/2 liberates their respective binding partners, facilitating preinitiation complex formation and initiating translation [39,40].

In TS, a loss of function mutation in TSC1 or TSC2 leads to the hyperactivity of mTOR [41]. In animal models of TS, the hyperactivation of mTORC1 by TSC1 or TSC2 ablation increases cell size, cell survival, and the production of slow-growing tumors, which is consistent with unrestrained protein translation [42,43,44]. In addition to protein translation, mTORC1 activation also dampens the levels of autophagy via ULK1 [45]. Autophagy plays a critical role in the degradation and recycling of cellular components. Deletion of TSC2 increases both AMPK and mTORC1 inhibition of autophagy, thus promoting tumorigenesis in TS patients [46,47].

Both mTOR complexes share the binding partners DEPTOR and mLST8 in common; however, the rapamycin-insensitive Rictor is unique to mTORC2. Upstream regulators of mTORC2 are not well defined; however, growth factor signaling is known to activate mTORC2 [48]. Like mTORC1, there are multiple downstream targets of mTORC2, including PKC, SGK1, MST1, and Akt. Through PKC and MST1, mTORC2 can increase cell survival and proliferation [49]. Activation of mTORC2 results in phosphorylation of protein kinase B/Akt at serine 473, which, in turn, positively regulates mTORC1 activity [48,50]. Akt is a key regulator of survival during cellular stress, and dysfunction can promote the development of tumors. The mTORC2 binding partner mSin1 localizes the complex to the plasma membrane near Akt [51]. Deletion of the pleckstrin homology (PH) domain of mSin1 leads to constitutive mTORC2-AKT signaling. Recent studies have shown that the PH domain of mSin1 can inhibit mTORC2 complex formation and reduce Akt signaling [52]. Akt has several downstream targets that can affect both transcription and protein translation. Akt phosphorylates both forkhead box class O (FOXO) transcription factors and MDM2, leading to increased transcriptional activity and cell survival. Additionally, TORC2 activation in yeast, via Ypk1, regulates G2/M cell cycle entry, as well as actin organization and endocytosis [53,54].

## 4. mTOR Complexes in Cell Cycle Regulation

Proper development requires the maintenance of cell growth and cell cycle progression in stem cells, factors that are mediated by mTOR [55,56,57]. Insulin and growth factor signaling pathways activate PI3K through tyrosine kinase receptor activation. PI3K, in turn, stimulates Akt activation [58]. Akt also leads to GSK-3 phosphorylation, which allows for β-catenin and cyclin D1 activation to promote transcription and cell cycle progression [59,60]. There are three mammalian homologous isoforms of Akt. While the functions of each isoform overlap, it has been reported that Akt1 and Akt3, in particular, contribute to cell survival and nervous system development [61,62,63]. Increased levels of both Akt and mTOR have been observed in tumors; however, driving mTORC1 activity alone is not sufficient for cancer progression in cells due to the possible negative feedback on Akt from downstream mTOR targets [56].

The effects of mTOR activation on cell cycle and cell growth vary during the developmental time points. Blastocyst-stage mouse embryos that are treated with rapamycin display decreased trophoblast proliferation without affecting the pluripotent inner cell mass cells [64]. During the gastrulation stage, rapamycin treatment can reduce proliferation rates in primary germ layers. However, complete deletion of the kinase domain of mTOR shortly after implantation leads to embryonic lethality [64,65]. Additionally, the role of mTOR on cell cycle progression could be mediated through positive neurometabolic-vascular coupling in an increased number of G1–S cycling cells in the SVZ [66]. These data illustrate the importance of mTOR interaction amongst early developmental lineages and timing specificity.

Akt activity can regulate cell cycle progression, a process that is essential to the maintenance or the differentiation of stem cells. Levels of cyclin D1 is required for progression through G1 and could lead to complete mitosis [67]. Akt has been shown to stabilize the cell cycle regulatory protein p21Cip1/WAF1 through phosphorylation, which inhibits proliferating cell nuclear antigen (PCNA) and further promotes the assembly of the cyclin D1: cyclin-dependent kinase 4 (CDK4) complex [68]. Cell growth is reliant on nutritional availability and sensing environmental cues, mediated by insulin signaling to mTORC1 and further downstream signaling to S6K1 and 4E-BP1. The downstream effect of these signaling when nutrient and growth factor availability is high is the promotion of translation initiation [69].

Previous studies have shown that the alteration of upstream elements in mTORC1 signaling can lead to the enhanced proliferation in NSCs [70]. Conditional deletion of PTEN not only increases soma size and aberrant migration, but also increases cortical thickness from overproduction of neurons [71]. Deletion of PTEN in human neural progenitors cultured in brain organoids enhances proliferation and can stimulate cerebral folding [72]. UTX, a histone demethylase, promotes PTEN expression, and deletion of UTX during neural development enhances Akt and mTOR phosphorylation, increasing NSC proliferation [73]. Overexpression of Akt1 can induce proliferation in cortical progenitors [74]. Inhibition of mTORC1 activity in PTEN conditional knockout mice rescues soma hypertrophy and seizure frequencies [75,76]; however, mTORC1 inhibition only partially reverses morphological abnormalities, suggesting that Akt activation by PTEN deletion may exert pathological effects independent of mTORC1 [77]. These results suggest that cell proliferation is enhanced by Akt activation via mTORC1-independent mechanisms.

**Figure 2:**
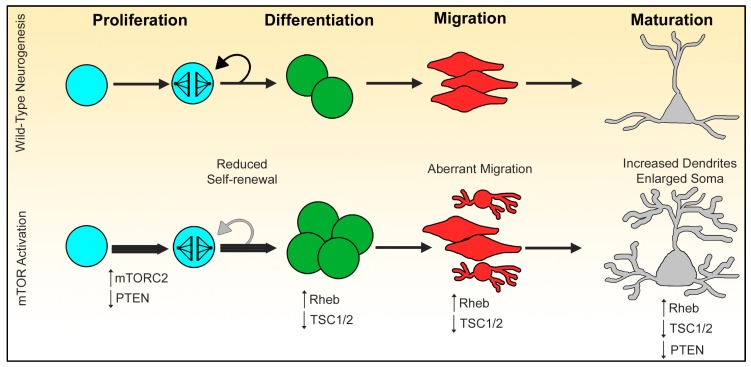
Effects of mTOR activation during neurogenesis. Neural stem cells (blue) undergo proliferation and either give rise to more stem cells (self-renewal) or daughter cells (green, differentiation). Activation of mTORC2 promotes neural stem cells (NSC) cell cycle entry through Akt. Hyperactivation of mTORC1 results in diminished self-renewal, favoring differentiation and lineage expansion. Daughter cells then migrate (red) from proliferation zones to their terminal positions. Activation of mTORC1 results in aberrant migration of daughter cells. Upon reaching their terminal positions, newly born neurons (gray) extend neurites and properly form dendritic arbors. Cells with high levels of mTORC1 activity can severely alter dendrite formation and synaptic integration. Upward pointing arrows indicate increased activity of designated genes or proteins. Downward pointing arrows indicate decreased activity or knockdown of designated genes or proteins.

Although fewer studies have examined mTORC2 in NSC physiology, recent research has shown a role for mTORC2 in promoting NSC proliferation. IGF1 induces PI3K and mTORC2 activation in cortical progenitors, promoting proliferation [78]. Phosphorylation of Akt at S477 and T479 residues by mTORC2 promotes Akt activation and cell survival and cell cycle entry [79]. Notch is a key regulator of NSC self-renewal, proliferation, and differentiation [80]. Previous studies have shown that non-canonical notch signaling activates mTORC2/Akt signaling cascade, promoting both cell proliferation and tumor formation [81,82]. Recent studies have shown that the activation of mTORC1 at targets downstream of Akt do not promote NSC cell cycle entry [43,83,84]. Taken together, these studies suggest that mTORC2 can promote NSC proliferation through Akt activation, but mTORC1 activation alone is insufficient to promote cell cycle entry (Figure 2).

## 5. Activation of mTOR Complexes Promotes Neural Stem Cell Differentiation

The role of the two mTOR complexes vary in stem cell maintenance due to their different downstream targets. mTORC1/p70S6K is maintained at lower levels than mTORC2 in embryonic stem cells (ESCs) through TSC1/2 activation [85]. ESCs display reduced global translation rates and subsequently lower p70 S6K to maintain their undifferentiated state. Elevations in mTORC1 signaling through knockdown of TSC2 display an increase in protein synthesis and loss of stemness [85]. Complete blocking of global translation resulted in cell death, which suggests that mTORC1 is essential in low signaling environments [85]. The function of the higher levels of mTORC2 is not as clear but thought to be related to cytoskeletal functioning and its interaction with Akt [85]. Additionally, DEP domain-containing mTOR interacting protein (DEPTOR), which is an endogenous mTOR inhibitor, is upregulated in undifferentiated stages. The role of mTORC1 inhibition through DEPTOR has been shown as a critical factor in the maintenance of stemness, and it is downregulated upon differentiation [86].

Disrupting mTOR signaling during cortical development presents pathological defects. mTORC1 is critical for NSC differentiation into daughter cells, as reduced activity reduces neural progenitor cell populations [87]. This regulation is accomplished by the targeting of the translational repressor 4E-BP2 [83]. Additionally, insulin signaling promotes neuronal differentiation in NSCs via mTORC1 activation [88]. Deletion of Raptor in cortical progenitors results in microcephaly presenting around E17.5, indicating a strong role for mTORC1 in maintaining proper cell size and number. In this study, dense cortical layering was observed when compared to controls, but the positioning of neurons was not disturbed. Thus, the loss of Raptor is suggested to be specific to differentiation and migration in corticogenesis [89]. Furthermore, TSC1 deletion and Rheb activation promotes the differentiation and the misplacement of cortical progenitors in the embryonic brain [43,90]. Additionally, the role of mTORC2 in corticogenesis could be perturbed by the deletion of Rictor in development. Rictor deletion resulted in smaller cell size, yet not as profound as Raptor deletion, and altered neuron morphology when limited to purkinje cells [91]. Normalizing protein translation of hyperactive mTORC1 by blocking mTORC1-dependent phosphorylation of 4E-BPs is sufficient to prevent neuronal misplacement and cell enlargement [90].

In the first week of postnatal life, the embryonic ventricular zone (VZ) transitions to the adult SVZ with mTOR activity driving self-renewal or differentiation. The proliferation of RGCs slows and these precursors differentiate into astrocytes, ependymal cells, and adult NSCs in the SVZ. NSCs give rise to TACs that give rise to neuroblasts [66]. Forming a chain-like arrangement, the neuroblasts migrate through the rostral migratory stream to the olfactory bulb [25]. Postnatal and adult NSCs in the SVZ predominantly give rise to olfactory bulb interneurons and also some oligodendrocytes in corpus callosum, fimbria, and striatum [92]. mTORC1 activity in the SVZ is linked to neural lineage expansion, resulting in proliferative cells and newborn neuroblasts. Hyperactivation mTORC1 has been shown to induce symmetric differentiation of NSCs into proliferative daughter cells [84]. Sustained activation of mTORC1 by TSC1 deletion eventually leads to a reduction of proliferative stem cells in the SVZ [43]. This effect appears to be mediated directly by cap-dependent translation as knockdown of 4E-BP2 promotes the differentiation of NSCs into daughter cells [83]. Based on these studies, mTORC1 control of cap-dependent translation modulates the balance between self-renewal and differentiation in NSCs.

## 6. Hyperactivation of mTORC1 Results in Aberrant Migration

In the VZ, RGCs are responsible for generating the vast majority of excitatory and projections neurons of the cortex [93,94]. Proliferation of RGCs eventually produce intermediate progenitor cells that migrate into the SVZ and typically undergo at least one round of cell division to produce immature neurons [95,96]. The generation of Tbr2+ intermediate progenitors is sustained by mTORC1 activation [97]. In addition to intermediate progenitors in the SVZ, populations outer radial glial cells undergo successive rounds of division and contribute to the expansion of the neocortex in primates [98,99]. As additional neurons arise, they migrate into the preplate to form the cortical plate. Neurons that are generated in the proliferative zones of the cortex abruptly change in shape and direction as they populate the cortical plate [17]. Additional migrating neurons arrive in the cortical plate, bypassing earlier-generated neurons to form the cortical layers in an inside-out sequence [100]. Inhibitory GABAergic interneurons that populate the cortex arise from proliferation zones in the ventral ganglionic eminences, and tangentially migrate into the cortex [101]. These neurons migrate long distances that are guided by chemical cues in the external environment, which are likely secreted from endothelial cells of the developing vasculature [102].

The PI3K-Akt-mTOR pathway plays an essential role in the proper development of cortical layers and differentiation. Hyperactivation of this axis recapitulate pathology present in developmental disorders. Mutations of mTOR result in cortical delamination and dysmorphic neurons [103]. The formation of tubers and micro nodules are the most pronounced symptoms associated with mTOR pathway dysregulation. Enhanced mTOR activity in TS often leads to cortical malformations during development, slow-growing astrocytomas, and cognitive deficits [6]. Mouse models of TS exhibit similar aberrant migration during cortical development, enhancing the cortical thickness and tuber-like lesions [42,43,104]. Rapamycin treatment in animal models of cortical dysplasia rescue these cortical aberrations [61]. The conditional deletion of mTOR in progenitors of the ganglionic eminences led to a reduction in overall interneurons in the cortex [105]. Disrupted-in-Schizophrenia 1 (DISC1) suppresses Akt and mTOR activation, negatively regulating neuron development and migration in the adult hippocampus [106]. In addition, DISC1 knockdown reduces tangentially-migrating interneurons, reaching the embryonic cortex [107,108].

In the postnatal SVZ, NSCs give rise to migrating neuroblasts that navigate long distances to reach the OB. Upon reaching the OB, these newly born neurons tangentially migrate from the RMS to reach their final destinations in the granule and glomerular layers [109]. Deletion of TSC1 in postnatal NSCs leads to aberrant migration and heterotopias, mirroring results that were observed in embryonic mutations [110]. Similarly, the constitutive activation of Rheb enhances neurogenesis, producing greater numbers of neurons in the OB, while also generating ectopic neuronal differentiation in the RMS and olfactory micronodules [111]. Taken together, studies of both embryonic and postnatal neurogenesis reveal a critical role for mTORC1 in regulating the migration of newly born neurons to their proper destinations.

## 7. Role of mTORC1 in Neurite Development and Synapse Formation

Activation of mTOR complexes exerts profound effects on all the processes of neurogenesis, including dendrite formation. NSCs give rise to diverse populations of neurons, each of which had dendritic trees with disparate morphologies. The dendritic arborization established during maturation determines how the neuron integrates thousands of synaptic inputs during its existence. Although individual neuronal subtypes have specific programs during the formation of the dendrites, there are common features and steps that are shared by all populations of neurons. Upon reaching their terminal position, neurons extend dendrites away from the soma towards their target are guided by external signals. Next, dendrites grow in length and diameter, and they develop the branching characteristic of their subtype. Third, dendritic growths are spatially restricted by repulsive signals [112]. Fourth, dendrites typically start to form specialized structures, such as dendritic spines to enhance synaptic communication. Finally, dendrites and spines are pruned through retraction and elimination to form mature circuits [113].

Upon reaching the cortical plate, all of the excitatory cortical neurons share a similar morphology, which is a single apical dendrite that branches within layer 1 of the cortex. Over time, basal dendrites sprout from the soma and the apical dendrite branches. Dendritic differentiation only occurs after cells completed their migration. PTEN deletion enhances mTOR signaling and increases dendritic aborization in cortical neurons [77]. Similarly, PTEN deletion in hippocampal granule neurons increased dendritic branching and synaptic excitation [114]. In addition, driving mTORC1 via TSC1 knockout or Rheb activation also produces greatly enhanced dendritic branching [42,115]. Inhibition of cap-dependent translation through the overexpression of 4E-BP1 can partially rescue mTORC1-driven dendritic hypertrophy [90].

Granule neurons comprise the largest population of interneurons in the olfactory bulb. Their soma resides in the granule cell layer, and they extend both basal dendrites and a single apical dendrite that branch extensively in the external plexiform layer. Granule neurons are unique in that they are anaxonic, forming dendrodentritic synapses via spiny processes [116]. Several studies have shown that mTORC1 activation increases dendritic complexity in olfactory granule neurons. Postnatal deletion of TSC1 drives dendritic hypertrophy in granule neurons [110,117]. Moreover, Rheb activation increases dendritic branching of granule neurons, particularly basal dendrites [111,118]. Interestingly, the knockdown of global mTOR inhibits apical dendrite branching, but mTORC1-specific inhibition does not reduce dendrite complexity [119]. These results suggest that different mTOR complexes could regulate the apical and basal dendritic trees individually.

Sprouting from newly established dendrites, filopodia begin to emerge during synaptogenesis and form new synapses with nearby neurons. During this period of synaptic development, filopodia can recruit axons, making contacts that drive morphological changes in the young spine. Pruning occurs as synaptic activity matures, thus reducing the overall density of spines. TSC1/2 deletion in neuronal cultures show reduced spine formation with the remaining spines demonstrating an immature filopodia morphology [120,121]. These results were not duplicated when being tested in the postnatal hippocampus [122]. In addition, studies in the animal models of TS have not reached a clear consensus [14,90,123]. However, TSC2 heterozygous mice do display an increase in dendritic spine density at 1 month of age. As removal of unnecessary spines begin around three weeks following birth, this result could reflect a deficit in pruning rather than increased formation of filopodia-like projections. Indeed, rapamycin treatment rescues spine elimination in TSC2 heterozygous mice by enabling autophagy [124]. Reduction in autophagy could disrupt the synaptic transmission. Deletion of Atg7 decreases neurotransmission and ASD model mice have disrupted synaptic plasticity that is associated with glutamate signaling [125,126].

Long-term maintenance and the adaptability of the synapse is crucial for neuron survival and function. Long-term synaptic plasticity requires the expression of new proteins, which are produced both in the soma and locally in the dendrite. Activation of mTORC1 is central to the regulation of translation initiation in the dendritic spine. Glutamate signaling through mGluR and N-methyl-D-aspartate (NMDA) receptors upregulates mTORC1 activation, suggesting an important role for mTOR in synaptic plasticity [127,128]. While mTORC1 activation alone seems to be insufficient to alter dendritic spine shape and function, paired activation of synaptic stimulation, and mTORC1 increases synapse volatility [129]. Increased activation at synapses resulting from mTORC1 dysregulation has been observed in mouse models of TS [42,43]. Additionally, epilepsy is prevalent in patients with TS, and more than 50% of patients are comorbid with ASD. Hyper-connectivity of neuronal circuits by disruption in protein synthesis is thought to drive behavior and cognitive impairment in autism patients [7]. Additionally, a mouse model presented ASD-like behavioral deficits with hyperactivity of mTOR through TSC1/2 mutations. Rapamycin administration was successful in ameliorating the behavioral and the pathological phenotype in the mutant mice [15]. Dysregulation of mTORC1 appears to be integral to the formation and maintenance of synapses, foundational dysfunction that can result in epilepsy and ASD.

## 8. Conclusions

Throughout development of the nervous system, mTOR plays crucial roles in proliferation, differentiation, and neurite outgrowth and synaptic formation. Many of the recent studies into the role of the mTOR complexes have focused on mTORC1 and control of cap-dependent translation. Proper regulation of protein translation is critical in maintaining self-renewal of stem cells, dendrite formation, and synaptic plasticity. While good evidence exists that mTORC2 phosphorylates Akt, very few studies have demonstrated upstream regulators of mTORC2. Even fewer in vivo studies have examined the role of mTORC2 in neurogenesis. Future studies are needed to elucidate the role of mTORC2.

Regulation of synaptic development and activity is critical for circuit formation and function. To date, the disruption of mTOR signaling has resulted in varying degrees of dendrite alterations. Many studies have demonstrated that activation of mTORC1 leads to increased dendrite length and complexity, but there is still conflicting evidence regarding spine formation and density. These disparities could be due to differences in method of mTOR activation, neuronal subtype, or temporal activation of mTOR. More studies are needed to understand the mechanisms that control mTOR complexes during synapse formation. The mTOR complexes are essential to neurogenesis and the establishment of neural circuits. A multitude of external signals and transduction pathways modulate mTOR activity. Understanding the role of these kinase complexes is necessary to developing future therapies for current neurodevelopmental disorders.

## Abbreviations

4E-BP1	Translation initiation factor 4e binding protein
4E-BP2	4E- protein phosphatase 2
Akt	Protein Kinase B
ASD	Autism Spectrum Disorder
CDK4	Cyclin-dependent kinase 4
DEPTOR	DEP domain-containing mTOR interacting protein
DISC1	Disrupted-in-Schizophrenia 1
EGF	Epidermal growth factor
eIF-4e	Eukaryotic translation initiaton 4e
Erk	Extracellular signal related kinase
ESC	Embryonic stem cells
FOXO	Forkhead box class O
Gsk-3β	Glycogen synthase kinase 3 beta
IGF	Insulin-like growth factor
MDM2	Mouse double minute 2 homolog
MLST8	mTOR associated protein LST8
mSin1	Mammalian stress-activated protein kinase-interacting protein
mTOR	mammalian target of rapamycin
mTORC1	mammalian target of rapamycin complex 1
mTORC2	mammalian target of rapamycin complex 2
NMDA	N-methyl-D-aspartate
NSC	Neural stem cell
OB	Olfactory bulb
P21Cip1/WAF-1	Cyclin dependent kinase inhibitor 1
PCNA	Proliferating cell nuclear antigen
PDK1	Phosphoinosidite-dependent kinase 1
PH PI3K	Pleckstrin homology Phosphoinositide 3 kinase
Pras40	Proline-rich AKT substrate
PTEN	Phosphatase and tensin homolog
Raptor	Regulatory-associated protein of mTOR
RGC	Radial glial cell
Rheb	Ras homolog enriched in brain
Rictor	Rapamycin-insensitive companion of mTOR
RMS	Rostral migratory stream
S6k1	S6 Kinase 1
SVZ	Sub ventricular zone
TAC	Transit amplifying cell
Tbr2	T-box brain protein 2
TS	Tuberous sclerosis
TSC1	Tuberous sclerosis complex 1
TSC2	Tuberous sclerosis complex 2
UTX	Ubiquitously transcribed tetratricopeptide repeat, X chromosome
VZ	Ventricular zone
Ypk1	Yeast serine/threonine protein kinase 1
